# Lung Mesenchymal Stem Cells Ameliorate Elastase-Induced Damage in an Animal Model of Emphysema

**DOI:** 10.1155/2018/9492038

**Published:** 2018-03-14

**Authors:** Donato Cappetta, Antonella De Angelis, Giuseppe Spaziano, Gioia Tartaglione, Elena Piegari, Grazia Esposito, Loreta Pia Ciuffreda, Angela Liparulo, Manuela Sgambato, Teresa Palmira Russo, Francesco Rossi, Liberato Berrino, Konrad Urbanek, Bruno D'Agostino

**Affiliations:** Department of Experimental Medicine, Section of Pharmacology, University of Campania “Luigi Vanvitelli”, Naples, Italy

## Abstract

Pulmonary emphysema is a respiratory condition characterized by alveolar destruction that leads to airflow limitation and reduced lung function. Although with extensive research, the pathophysiology of emphysema is poorly understood and effective treatments are still missing. Evidence suggests that mesenchymal stem cells (MSCs) possess the ability to engraft the injured tissues and induce repair via a paracrine effect. Thus, the aim of this study was to test the effects of the intratracheal administration of lung-derived mouse MSCs in a model of elastase-induced emphysema. Pulmonary function (static lung compliance) showed an increased stiffness induced by elastase, while morphometric findings (mean linear intercept and tissue/alveolar area) confirmed the severity of alveolar disruption. Contrarily, MSC administration partially restored lung elasticity and alveolar architecture. In the absence of evidence that MSCs acquired epithelial phenotype, we detected an increased proliferative activity of aquaporin 5- and surfactant protein C-positive lung cells, suggesting MSC-driven paracrine mechanisms. The data indicate the mediation of hepatocyte growth factor in amplifying MSC-driven tissue response after injury. Our study shed light on supportive properties of lung-derived MSCs, although the full identification of mechanisms orchestrated by MSCs and responsible for epithelial repair after injury is a critical aspect yet to be achieved.

## 1. Introduction

Chronic obstructive pulmonary disease (COPD) and pulmonary emphysema, together with asthma, are highly prevalent lung diseases worldwide [1, 2]. These disorders are characterized by airflow limitations, airway inflammation, and hyperresponsiveness [3, 4] and can be correlated with other pathologies as well [5, 6]. In particular, pulmonary emphysema is defined as a progressive disease related to cigarette smoking and other respiratory insults, resulting in permanent enlargement and loss of alveoli and bronchioles [7]. The chronic inhalation of irritants attracts inflammatory cells and inflammatory mediators into the lungs, where they impair protease-antiprotease balance thus leading to the destruction of alveolar units [8]. Given the irreversibility of this disease and the significant number of deaths and the short-lasting benefits of current therapies, the understanding of mechanisms underlying lung tissue homeostasis is critical for developing new therapies aiming at replenishing diseased alveoli. Thus, lung regeneration biology is an area of intense studies in the search for new strategies for combating human lung diseases.

A significant body of evidence has demonstrated that mesenchymal stem cells (MSCs), harvested from adult organs such as bone marrow and adipose tissue and administered in the damaged tissue, may induce organ repair mainly through paracrine effects [9, 10]. Despite conflicting results about the degree of engraftment of MSCs, it seems clear that these cells may be protective against tissue injury independently from their ability of engraftment [11, 12]. The usefulness of MSCs, whose benefits are also linked to their low immunogenicity [13], has been proven in various models of pulmonary diseases [14, 15]. In particular, MSCs from bone marrow and adipose tissue had a therapeutic effect in an experimental cigarette smoke-induced and elastase-induced emphysema models [16–19]. To date, several clinical trials have investigated the safety and feasibility of MSC administration reporting no serious adverse events although indicating modest effects in spite of undoubted efficacy in animal studies [20, 21].

Most studies have focused attention on bone marrow- and adipose tissue-derived MSCs to assess their potential on lung diseases, and the orientation of scientific community for these sources is essentially dictated by the readiness to obtain MSCs from these sites. On the other hand, little is known about the biological significance of lung-derived MSCs also because of obvious difficulties to obtain lung biopsies that have limited the studies on these cells. Nonetheless, lung MSCs may be relevant in alveolar homeostasis and repair after injury and may need consideration as a potential tool or target for cell-based therapy that involves other pulmonary cell populations. Therefore, the aim of our study was to test the effects of intratracheal administration of pulmonary MSCs into elastase-injured emphysematous lungs. In contrast to the majority of studies that utilized the systemic administration of cells, in our work, the intratracheal delivery was used. This route provides benefits over a systemic infusion, such as the reduction of cell number and the low risk to engraft other organs.

## 2. Materials and Methods

### 2.1. Cell Isolation and Culture

Six/eight lungs were harvested from 2-month-old male C57BL/6J mice (Charles River Laboratories) for each isolation of murine lung-derived MSCs. Samples were collected in 100 mm diameter culture dishes and were quickly washed with DPBS w/o Ca^2+^ and Mg^2+^ (Euroclone) to wash out the blood. Large vascular and bronchial components were removed as well. In order to obtain a cell suspension, the lungs were tinily minced and enzymatically dissociated with a prewarmed collagenase solution [280 U/ml type II collagenase (Worthington), 100 U/ml penicillin, and 100 *μ*g/ml streptomycin (pen/strep, Euroclone)]. After a 45 min digestion at 37°C under agitation, collagenase was deactivated by adding a double volume of precooled quenching buffer [0.5% bovine serum albumin (Sigma-Aldrich); pen/strep]. Cell suspension was further purified by several passages through cell strainers [70 and 40 *μ*m pores (BD Biosciences)] and centrifuged at 1200 rpm for 10 min to remove debris. Cell pellet was collected and then washed with DPBS. After centrifugation (1200 rpm for 10 min), the cell pellet was plated in 60 mm diameter culture dishes. MSCs were selected by adhesion. After removal of nonadherent cells, cells were cultured in *α*-MEM supplemented with 10% FBS and pen/strep and seeded at a density of 7 × 10^4^ cells/cm^2^. The cells were used from passage 3.

### 2.2. Flow Cytometry

FACS analysis was performed for MSC phenotype characterization, and 10,000 cells were detected for each surface marker. PE-conjugated antibodies for CD14, CD34, CD44, CD45, CD73, CD90, and CD105 were used (BD Biosciences). Isotype control was utilized to define the threshold for each specific signal. Data were acquired by FACSAria (BD Biosciences) and analyzed by FCS Express 6 (De Novo Software).

### 2.3. GFP Lentiviral Infection

MSCs were transduced with a Cignal Lentivirus carrying GFP and puromycin resistance genes at a MOI of 50 (Qiagen). After 24 h, cells were washed and transduction medium was replaced by fresh medium. At this point, Cignal reporter constructs were integrated into the genomic DNA of target cells. To select the cells that stably expressed the GFP reporter gene, puromycin (5 *μ*g/ml) selection was performed for one week. GFP-positive MSCs were collected, diluted in fresh medium, and used for *in vivo* procedures.

### 2.4. Animal Housing

The experimental protocol was approved by the Animal Care and Use Committee of the University of Campania “Luigi Vanvitelli” (294/2016-PR 24.03.2016). Animal care complied with Italian regulations on the protection of animals used for experimental and other scientific purposes (116/1992) as well as with the EU guidelines for the use of experimental animals (2010/63/EU). Mice were housed in the Animal Facility of the University of Campania “Luigi Vanvitelli.” Food and water were supplied *ad libitum*. Room temperature was set at 22°C–24°C, relative humidity at 40%–50%, and the day/night cycle at 12 h/12 h. In order to prevent any possible animal pain, all experimental animals were anesthetized with ketamine and medetomidine hydrochloride. All mice were sacrificed by cervical dislocation.

### 2.5. Experimental Protocol

Emphysema was induced in 2-month-old female C57BL/6J mice by intratracheal administration of porcine pancreatic elastase (PPE; 80 U/kg in 100 *μ*l of PBS on day 0). Mice were then randomized into two experimental groups: (1) PPE-MSCs (*n* = 18), receiving lung MSCs (5 × 10^4^ cells in 50 *μ*l medium per animal) and (2) PPE (*n* = 18), receiving standard cell medium. MSCs or medium was intratracheally administered on day 21. Naïve mice (*n* = 18), not subjected to any treatment, served as the control. BrdU was injected twice a day (50 mg/kg, i.p.) and added to the drinking water (1 mg/ml) in order to identify newly formed cells. All mice were sacrificed on day 31.

### 2.6. Intratracheal Administration

Prior to cell administration, mice were anesthetized with ketamine (40 mg/kg, i.p.) and medetomidine hydrochloride (0.15 mg/kg, i.p.). A 20-gauge custom-made catheter was inserted into the trachea via the mouth and connected to a mouse ventilator (Harvard Apparatus). After checking the correct placement of the catheter, the ventilator was disconnected and the delivery of the necessary vehicle (PPE, MSCs or medium) was carried out by using a syringe with a fine needle. Then, mice were mechanically ventilated for 3 min and placed in a warm chamber until they recovered consciousness (5–15 min).

### 2.7. Static Lung Compliance

After animal sacrifice, the body cavity was opened, an incision was made in the trachea, and a 20-gauge catheter was inserted and secured with a suture. Static lung compliance was measured with a 5 cc syringe connected to the trachea via a catheter and to a water manometer via a three-way stopcock. To get the inflation curves, 0.2 cc of air was manually injected, up to 3.0 cc. The resultant pressure from each incremental injection was read from the manometer approximately 1 s after the injection. Deflation was read in the same fashion, manually withdrawing 0.2 cc at a time, until reaching the maximum volume of 3.0 cc. The curves of inflation and deflation were measured twice for each animal. Volume was traced as a function of pressure. Static lung compliance was obtained through the average slope of each deflation curve at its midpoint [22].

### 2.8. Tissue Preparation

For histology, the lungs were perfused and fixed as previously described [23]. Tissue sections, 5 *μ*m in thickness, were used. For morphometric studies, sections were stained with hematoxylin/eosin (H&E, Sigma-Aldrich). For molecular biology analysis, the lungs were excised and subsequently stored at −80°C.

### 2.9. Morphometry

Morphometric assessment included the determination of the average interalveolar distance (mean linear intercept) and the calculation of tissue and airspace areas, corrected for the alveolar number. The mean linear intercept was measured by superimposing a grid over each image and counting the number of times the alveolar walls intercepted the grid lines. The equation

(1) $$\begin{array}{c} Mean\;linear\;intercept=\frac{\left(N\times L\right)}{m}, \end{array}$$

where *N* is the number of times the transverses were placed on the tissue, *L* is the length of the transverses, and *m* is the sum of all intercepts, gave mean linear intercept [22, 24]. Morphologic measurements were done with Image-Pro Plus software (Media Cybernetics).

### 2.10. Immunohistochemistry

Injected MSCs were detected by chicken polyclonal anti-GFP antibody (1 : 500, overnight at 4°C) (Abcam). Rat monoclonal CD45 (1 : 30, overnight at 4°C) (Novus Biological) was used to exclude the hematopoietic lineage in MSCs. Lung cells were identified by immunostaining for aquaporin 5 (AQP5; rabbit polyclonal, 1 : 100, overnight at 4°C) (Abcam) and surfactant protein C (SFTPC; rabbit polyclonal, 1 : 100, overnight at 4°C) (Santa Cruz Biotechnology). Cycling cells were visualized using mouse monoclonal anti-BrdU antibody (1 : 10, 1 h at 37°C) (Roche Diagnostics). The expression of hepatocyte growth factor (HGF; rabbit polyclonal, 1 : 100, overnight at 4°C) (Abcam) and its receptor c-Met (mouse monoclonal, 1 : 100, overnight at 4°C) (Cell Signaling) in the lung was also detected. Nuclei were stained with DAPI (Sigma-Aldrich). Secondary antibodies conjugated with FITC, TRITC, or Cy5 were used at the dilution of 1 : 100 for 1 h at 37°C (Jackson ImmunoResearch). The quantification of newly formed cells was performed by counting at least 200 AEC1 or AEC2 (*n* = 6 from each experimental group) and expressed as the percentage of BrdU-positive cells. Samples were analyzed with a Leica DM 5000B microscope a Zeiss LSM 700 confocal microscope.

### 2.11. Western Blotting

Tissue samples were homogenized in lysis buffer containing protease inhibitors (Sigma-Aldrich). Protein concentration was determined by Bradford assay (Bio-Rad Laboratories). 20 *μ*g of protein extracts was then separated by SDS-PAGE on 8–12% bis-acrylamide gel and transferred onto polyvinylidene fluoride membrane (Thermo Fisher Scientific). Membranes were probed with primary antibodies (1 : 1000 for 1 h at room temperature) against AQP5 (Abcam), SFTPC (Santa Cruz Biotechnology), HGF (Abcam), epidermal growth factor (EGF) (Elabscience), and vascular endothelial growth factor (VEGF) (Santa Cruz Biotechnology). Loading conditions were determined with glyceraldehyde-3-phosphate dehydrogenase (GAPDH; 1 : 20,000 for 1 h at room temperature) (Sigma-Aldrich). Peroxidase-conjugated secondary antibodies (Santa Cruz Biotechnology) were employed for primary antibody detection, antibody binding was visualized by enhanced chemiluminescence (1 : 10,000 for 1 h at room temperature) (Merck Millipore), and images were collected and analyzed using a Chemidoc-It Imager (Ultra-Violet Products). The optical density of the bands was measured with the Molecular Analysis software (Bio-Rad Laboratories).

### 2.12. Statistical Analysis

Results were reported as the mean ± SD. Statistics were performed by using GraphPad Prism (GraphPad Software). Significance among multiple comparisons was determined by the one-way ANOVA corrected with the Bonferroni's posttest. A value of *P* < 0.05 was considered significant.

## 3. Results

### 3.1. Immunophenotyping and Tissue Engraftment

**Figure 1:**
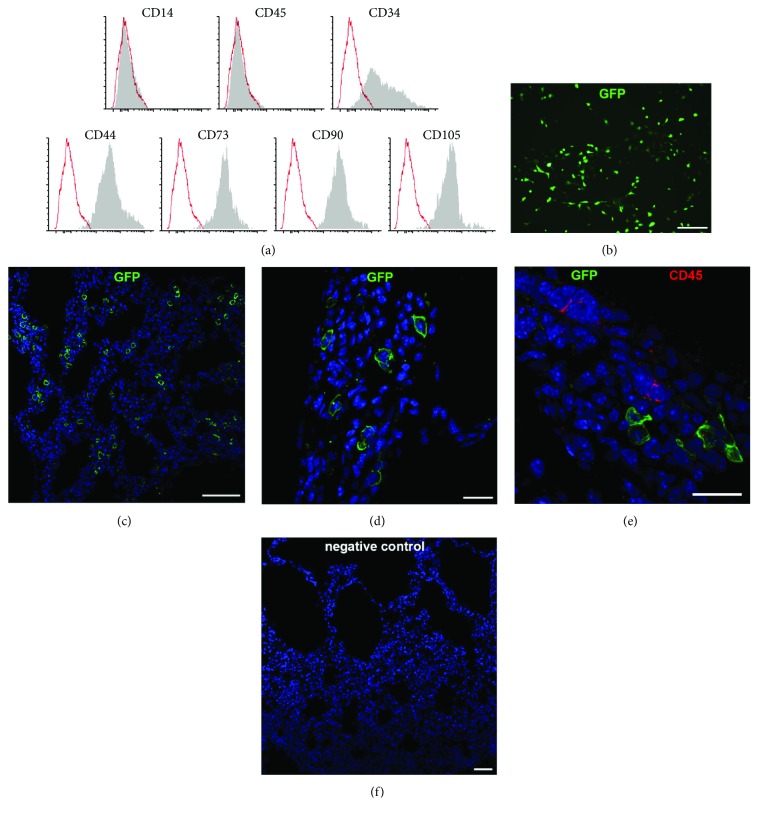
MSC characterization and engraftment. (a) Immunophenotypic profile by flow cytometry of MSCs isolated from adult mouse lungs. Grey-shaded peaks show CD markers; red histograms represent isotype control. (b) Representative image of MSCs after lentiviral transduction of GFP (green). (c, d) *In vivo* engraftment of GFP-positive MSCs (green) in emphysematous lungs ten days after intratracheal cell administration. (e) Representative image of GFP-positive MSCs (green) and CD45-positive cells (red). (f) Negative control for GFP staining in a PPE lung. Nuclei are counterstained with DAPI (blue). Scale bars: 20 *μ*m (d, e), 50 *μ*m (b, f), and 100 *μ*m (c).

The phenotype of MSCs isolated from the lung of healthy mice was addressed using flow cytometry. Lung-derived MSCs displayed the surface expression of CD44, CD73, CD90, and CD105, consistent with the profile of cells of mesenchymal origin. MSCs were also found to partially express the progenitor marker CD34 and to completely lack hematopoietic cell markers CD14 and CD45 (Figure 1(a)). After isolation and expansion, MSCs were infected with GFP-carrying lentivirus (Figure 1(b)). Immunofluorescence analysis addressing the capacity of MSCs to engraft within the injured tissue revealed the substantial presence of GFP-positive cells, negative for hematopoietic surface marker CD45, within pulmonary structures ten days after their administration (Figures 1(c)–1(f)).

### 3.2. Morphology, Morphometry, and Function

**Figure 2:**
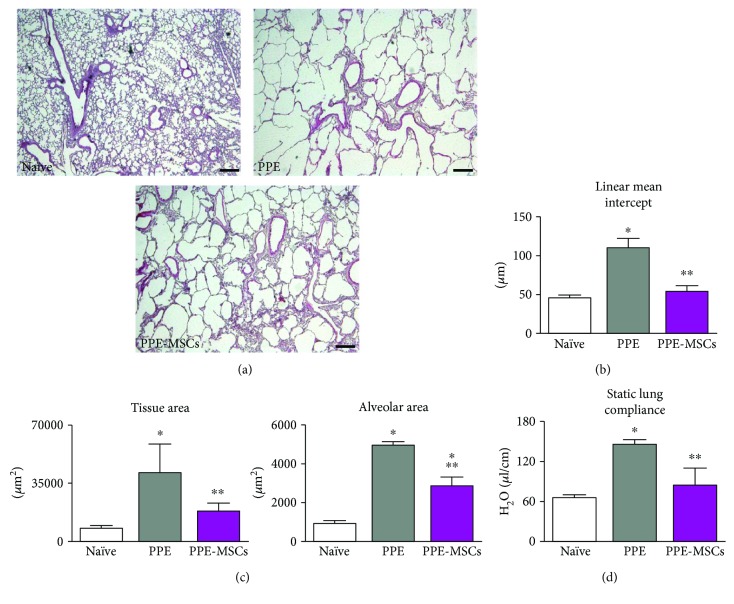
Lung histology and function. (a) Hematoxylin/eosin staining on lung tissue at day 31. (b) Morphometric analysis of the mean linear intercept. (c) Quantification of tissue and alveolar area per alveolus. (d) Functional measurements of static lung compliance. Data are expressed as the mean ± SD (*n* = 6 in each experimental group). Scale bars: 200 *μ*m. ^∗^*P* < 0.05 versus naïve; ^∗∗^*P* < 0.05 versus PPE.

Histological analysis revealed evident airspace enlargement and obliteration of the alveolar wall in the lungs injected with elastase. These changes were attenuated by the instillation of MSCs (Figure 2(a)). Quantification of alveolar destruction by the mean linear intercept showed a marked increase in the PPE group compared to naïve mice, while the treatment with MSCs induced a significant decrease (Figure 2(b)). The tissue area, alveolar area, and number of alveoli were used for the calculation of additional morphological parameters. The PPE group exhibited the increment of tissue and alveolar areas (normalized for alveolar number), consistently with the increment of the alveolar size. On the other hand, the administration of MSCs positively affected lung structures and partially reverted alveolar destruction observed in emphysematous mice (Figure 2(c)). To assess whether the altered histology was accompanied by changes in lung mechanics, static lung compliance was examined. Compliance, which significantly increased in the PPE group due to the poor elastic recoil, was instead reduced after the intratracheal administration of MSCs (Figure 2(d)).

### 3.3. Fate of Lung-Derived MSCs

**Figure 3:**
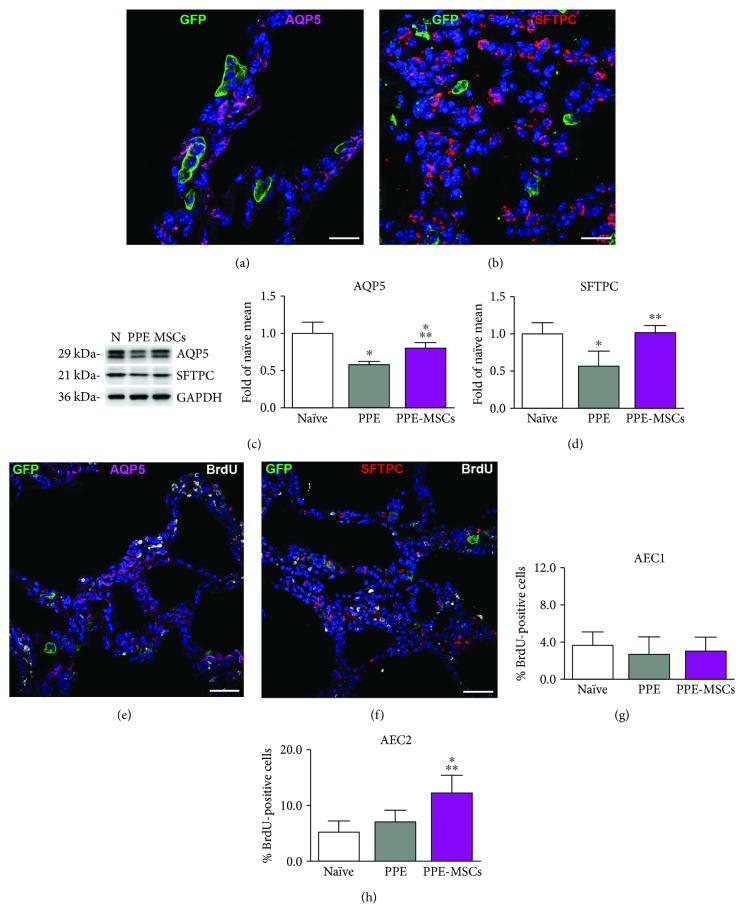
Biological effects mediated by MSCs. (a, b) Representative images of GFP-positive MSCs (green) lacking alveolar epithelial commitment in emphysematous pulmonary parenchyma. Alveolar type I (a) and type II (b) epithelial cells express aquaporin 5 (AQP5; magenta, pseudocolor) and surfactant protein C (SFTPC, red), respectively. (c, d) Protein expression of AQP5 (c) and SFTPC (d) in the lung by Western blotting. (e, f) Proliferative activity (BrdU; white, pseudocolor) in the PPE-MSC group; alveolar type I (e) and type II (f) epithelial cells expressed AQP5 (magenta, pseudocolor) and SFTPC (red), respectively. Scattered GFP-positive MSCs (green) are also present. Nuclei are counterstained with DAPI (blue). (g, h) Quantification of newly formed alveolar type I (g) and type II (h) epithelial cells. Data are expressed as the mean ± SD (*n* = 6 in each experimental group). Scale bars: 20 *μ*m. ^∗^*P* < 0.05 versus naïve; ^∗∗^*P* < 0.05 versus PPE.

To answer the question of how the presence of MSCs could participate to structural and functional changes observed in cell-treated mice, the *in vivo* fate of MSCs was examined. The engraftment of MSCs was not accompanied by a significant differentiation towards the pulmonary lineage, as confirmed by the lack of colocalization of GFP staining with both alveolar epithelial type I and type II cell (AEC1 and AEC2) markers, AQP5 and SFTPC, respectively (Figures 3(a) and 3(b)). Hence, excluding that, MSCs are able to acquire (at least to a significant extent) a pulmonary-committed phenotype which opens to the possibility that MSCs may indirectly orchestrate the activation of other cell types for the repair of tissue damage. Western blotting of elastase-treated lung tissue showed the reduced content of epithelial markers AQP5 and SFTPC, consistent with the destructive effect on the alveolar walls. The formation of new epithelium was suggested by the increased expression of AQP5 and SFTPC observed in the lungs receiving intratracheal administration of lung MSCs (Figures 3(c) and 3(d)). Indeed, in the PPE-MSCs group, a significant rate of proliferative cells confirmed the formation of new parenchyma. Interestingly, the analysis of alveolar epithelium revealed a higher proliferative rate of AEC2 (Figures 3(e)–3(h)).

### 3.4. Paracrine Action Induced by Lung-Derived MSCs

**Figure 4:**
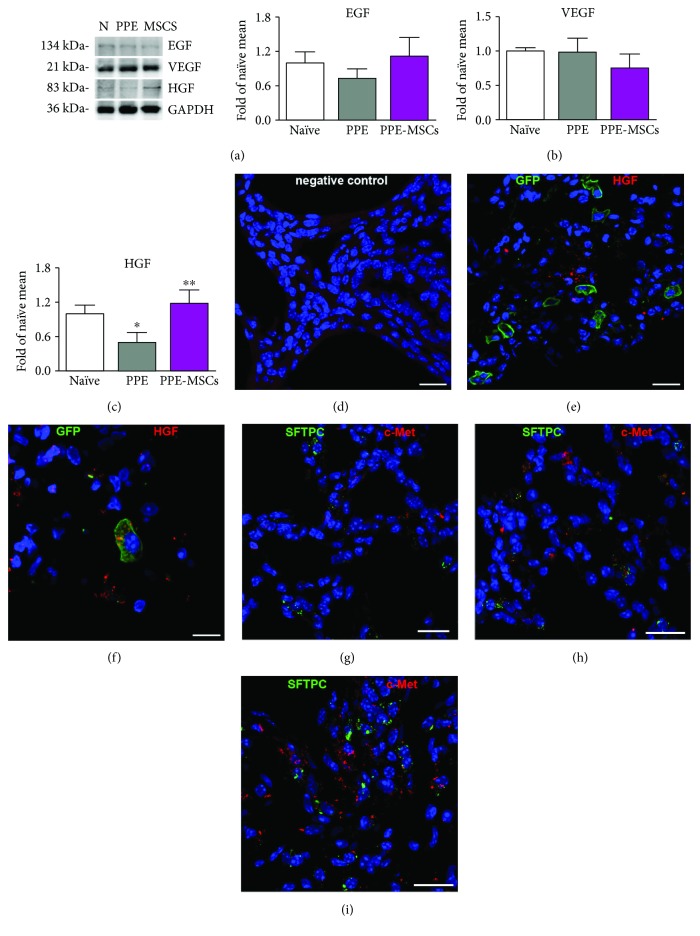
Growth factor profile and MSCs. (a–c) Protein expression of epidermal growth factor (EGF) (a), vascular endothelial growth factor (VEGF) (b), and hepatocyte growth factor (HGF) (c) in the lung by Western blotting. (d) Negative control for HGF staining. (e) Representative images displaying HGF (red) in the proximity of GFP-positive MSCs (green). (f) Intracellular content of HGF (red) in a GFP-positive MSC (green). (g–i) c-Met expression (red) in the lung parenchyma from the naïve (g), PPE (h), and PPE-MSC (i) groups; alveolar type II epithelial cells are shown by SFTPC (green). Nuclei are counterstained with DAPI (blue). Data are expressed as the mean ± SD (*n* = 6 in each experimental group). Scale bars: 10 *μ*m (f) and 20 *μ*m (g–i). ^∗^*P* < 0.05 versus naïve; ^∗∗^*P* < 0.05 versus PPE.

In the search for mechanistic insights that may drive repair and regenerative processes, we examined the expression of several growth factors such as EGF, VEGF, and HGF. The presence of these factors in the normal lung indicates their role in tissue homeostasis in physiological conditions. While Western blotting analysis revealed a nonsignificant modulation of EGF and VEGF, HGF expression, lower in PPE mice, was significantly boosted after the administration of MSCs (Figures 4(a)–4(c)). In situ analysis demonstrated the intra- and extracellular presence of HGF in the lung parenchyma of mice treated with MSCs. Moreover, GFP-positive cells presented scattered intracellular distribution of the growth factor (Figures 4(d)–4(f)). The importance of HGF signaling in the MSC-treated lungs was supported by the elevated content of its receptor c-Met that was also expressed by AEC2 (Figures 4(g)–4(i)).

## 4. Discussion

We report that intratracheal administration of lung-derived MSCs ameliorated alveolar damage induced by elastase. This effect may have been mediated by the release of HGF as MSC-dependent paracrine mechanisms. Activation of HGF/c-Met system, by promoting survival and proliferation of alveolar epithelial cells, may be a major determinant to trigger a reparative response in emphysema lung.

The elastase model “translates” major pathogenic mechanisms accounting for COPD: the protease-antiprotease imbalance, characterized by elevated production of proteases by inflammatory cells that determines the interruption of alveolar integrity [25]. In the present study, elastase-induced deterioration in lung function and structure was improved by lung MSCs, administered at the peak of airspace enlargement [24]. The partial recovery of microanatomy was reflected by the significant decrease of mean linear intercept and alveolar enlargement and the increase of the alveolar number. Some degree of regaining the mechanical performance measured by static lung compliance may be considered the causal consequence of the regenerative process upon alveolar units.

Phenotypic identity and plasticity of MSCs may depend on the tissue of origin and even vary within the same tissue. MSCs from multiple sources have the recurrent presence of mesenchymal markers and the concomitant absence of hematopoietic and endothelial markers. A number of different determinants, such as CD34, Sca-1, or CD117, may be expressed to different extents [20, 26]. Our data on lung-derived MSC phenotype confirmed the expression of the set of general MSC surface markers. Although the phenotype of MSCs across sources is similar, evidence shows a diverse behavior *in vivo* regarding the capacity to differentiate, migrate, or engraft. This implies that different gene expression or epigenetic signatures drive the cells to acquire distinct biological properties [27]. It is as much as logic that also the cell-receiving organ plays a pivotal role. Indeed, it has been demonstrated that lung-derived MSCs, when injected intravenously in a large number, possess a higher ability to engraft the lung with respect to bone marrow MSCs. Lung MSCs exhibited greater expression of genes encoding paracrine signaling and higher level of adhesive proteins [28].

MSC-related therapeutic potential in lung diseases incorporates two main mechanisms: immunomodulation and multilineage differentiation [10]. MSCs are immunosuppressive, and anti-inflammatory effects by means of cell-to-cell contact and the release of soluble factors modulate the activity of immune cells [29–31]. Data on MSC differentiation potential show mixed results, and the dispute whether this phenomenon occurs at tissue level is ongoing [32]. Studies have shown bone marrow MSCs possessing a low plasticity *in vivo* with a residual capacity to differentiate into either AEC1 or AEC2 likely dictated by their low engraftment [33]. In our hands, the engraftment of lung MSCs was not accompanied with a differentiation into endodermal lineage pointing that lung-derived MSCs are likely to coordinate the repair rather than directly replace lost tissue.

The mechanisms through which MSCs may modulate the function of other cells involved in tissue homeostasis remain largely unexplored. There is a consensus that factors such as VEGF, EGF, and HGF are involved in the protective and reparative effects of bone marrow and adipose MSCs [17, 33]. Similar to fetal development, when mesenchymal cells supply lung epithelial cells with trophic factors sustaining their growth, an interaction during the adult life is plausible [20]. The analysis of the growth factor profile has evidenced a stronger modulation of HGF, compared to EGF and VEGF. We have detected an elevation of the HGF/c-Met axis in the lungs of MSC-treated animals as well as HGF inside and in the proximity of GFP-positive cells. Moreover, administration of lung MSCs was accompanied by the proliferation of AEC2. In this regard, tissue repair observed in our study is consistent with a concept that a fraction of AEC2 may possess progenitor properties and, when activated, promote a repair of injured alveoli [34]. HGF is a potent morphogenetic and proliferative factor in case of injuries [35, 36] and stimulates epithelial cell proliferation in elastase-induced models, mediating alveolar formation and regeneration [16, 37, 38]. Human bone marrow MSCs, administered early at the onset of the emphysema, exerted anti-inflammatory and antiapoptotic effects mediated in part through MSC production of HGF [39]. Mice deficient in c-Met in alveolar epithelium exhibit impaired airspace morphology and reduced the number of surviving AEC2 [40]. Briefly, stimulation with HGF reversed airspace enlargement in the emphysematous lung, while *in vitro* experiments conducted on alveolar epithelial cells established protecting effects of HGF. The implication of HGF in mediating MSC-stimulated beneficial effects has also been demonstrated in experimental models of multiple sclerosis [41] further indicating a critical role for HGF and c-Met in the recovery from injuries.

## 5. Conclusions

We report previously unrecognized properties of adult mouse lung-derived MSCs that after local administration boost and orchestrate a local response to damage. Although several aspects of cellular physiology and *in vivo* behavior related to the therapeutic potential of lung-derived MSCs remain to be clarified, the comprehension of the mechanisms driving epithelial repair, as well as the interrelationships between epithelial cells and MSCs, may help to identify targets for pharmacological and/or cell-based interventions for lung diseases.
